# Cidofovir Activity against Poxvirus Infections

**DOI:** 10.3390/v2122803

**Published:** 2010-12-22

**Authors:** Graciela Andrei, Robert Snoeck

**Affiliations:** Laboratory of Virology and Chemotherapy, Rega Institute for Medical Research, KULeuven, Minderboredersstraat 10, B-3000 Leuven, Belgium; E-Mail: robert.snocek@rega.kuleuven.be

**Keywords:** cidofovir, poxviruses, acyclic nucleoside analog

## Abstract

Cidofovir [(S)-1-(3-hydroxy-2-phosphonylmethoxypropyl)cytosine, HPMPC] is an acyclic nucleoside analog approved since 1996 for clinical use in the treatment of cytomegalovirus (CMV) retinitis in AIDS patients. Cidofovir (CDV) has broad-spectrum activity against DNA viruses, including herpes-, adeno-, polyoma-, papilloma- and poxviruses. Among poxviruses, cidofovir has shown *in vitro* activity against orthopox [vaccinia, variola (smallpox), cowpox, monkeypox, camelpox, ectromelia], molluscipox [molluscum contagiosum] and parapox [orf] viruses. The anti-poxvirus activity of cidofovir *in vivo* has been shown in different models of infection when the compound was administered either intraperitoneal, intranasal (aerosolized) or topically. In humans, cidofovir has been successfully used for the treatment of recalcitrant molluscum contagiosum virus and orf virus in immunocompromised patients. CDV remains a reference compound against poxviruses and holds potential for the therapy and short-term prophylaxis of not only orthopox- but also parapox- and molluscipoxvirus infections.

## 1. Introduction

**Figure 1:**
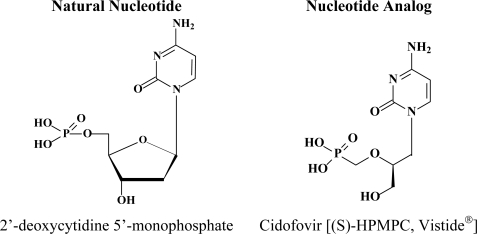
Chemical structure of (S)-1-(3-hydroxy-2-phosphonylmethoxypropyl)cytosine (HPMPC (CDV)) and its natural nucleotide.

The antiviral activity of (S)-1-(3-hydroxy-2-phosphonylmethoxypropyl)cytosine (HPMPC, cidofovir, CDV) (Figure 1) against human cytomegalovirus (HCMV) and other DNA viruses was first reported in 1986 [1]. In 1996, the intravenous form of CDV was licensed for clinical use, under the trade name of Vistide^®^, for the systemic treatment of HCMV retinitis in AIDS patients.

CDV is a close congener of HPMPA [(S)-9-(3-hydroxy-2-phosphonylmethoxypropyl)adenine], which was the first acyclic nucleoside phosphonate (ANP) described with broad spectrum anti-DNA virus activity [1,2]. This compound can be considered a hybrid between acyclic nucleoside analogs, such as (S)-9-(2,3-dihydroxypropyl)adenine (DHPA), which was previously described as an ANP with broad-spectrum antiviral activity, and a phosphonate analog such as phosphonoformic acic (PFA) or phosphonoacetic acid (PAA). PMEA [2-(phosphonylmethoxyethyl)adenine, adefovir] was developed in parallel with HPMPA, whereas HPMPC was derived from HPMPA by simply substituting a pyrimidine (cytosine) for the purine (adenine) moiety. Further modifications of the acyclic side chain of HPMPA led to PMPA [2-(phosphonylmethoxypropyl)adenine] and PMPDAP [2-(phosphonylmethoxypropyl)2,6-diaminopurine]. PMEA displayed potent activity against retroviruses (while maintaining activity against herpes- and hepadnaviruses) while the antiviral activity of PMPA was restricted to retro- and hepadnaviruses.

In regular nucleotides (or nucleoside phosphates), the phosphate group is attached through an ester bound (-P-O-C-) to the nucleoside. In the ANPs, the phosphate group—in a form of a phosphonate group—is already attached to the nucleoside analog, thus resulting in the formation of a phosphonomethyl ether (-P-C-O-), which unlike the phosphate ester linkage should resist any attack by esterases. Furthermore, the fact that a phosphonate group is built in the acyclic nucleoside skeleton turns these compounds in ANPs, and made it possible to bypass the first phosphorylation step. This first phosphorylation step, carried out by virus kinases, is necessary for the activation of the “classical” acyclic nucleoside analogs, such as acyclovir ACV and ganciclovir GCV.

The discovery of ANPs represented a breakthrough in the treatment of DNA viruses and retroviruses. According to their activity spectrum, the first generation of ANPs can be classified in three categories: (i) the “HPMP” (*i.e.*, 3-hydroxy-2-phosphonylmethoxypropyl) derivatives, represented by HPMPC (cidofovir, CDV), which displays activity against a broad variety of DNA viruses, (ii) the “PME” (*i.e.*, 2-phosphonylmethoxyethyl) derivatives with activity against DNA viruses and retroviruses, and iii) the “PMP” (*i.e.*, 2-phosphonylmethoxypropyl) derivatives, represented by, respectively, PMEA (adefovir) and PMPA (tenofovir). These three representative compounds have been licensed for the treatment of HCMV retinitis in AIDS patients (CDV, Vistide^®^), chronic hepatitis B virus infections (adefovir dipivoxil, Hepsera^®^) and HIV infections (tenofovir disoproxil fumarate, TDF, Viread^®^). TDF is also available in a fixed-dose combination form with emtricitabine (Truvada^®^) or emtricitabine and efavirenz (Atripla^®^) for the treatment of AIDS.

More recently, two new generations of ANPs have been synthesized. The second generation of ANPs includes the 2,4-diaminopyrimidine (DAPy) derivatives: HPMPO-DAPy [(R)-6-(3-hydroxy-2-(phosphonylmethoxy)propoxy)-2,4-diaminopyrimidine], PMEO-DAPy [6-(2-(phosphonylmethoxy)ethoxy)-2,4-diaminopyrimidine], and 5-X-substituted derivatives thereof, and PMPO-DAPy [(R)-6-(2-(phosphonylmethoxy)propoxy)-2,4-diaminopyrimidine] [3–5]. The third generation of ANPs comprises the ANPs containing as a base moiety 5-azacytosine; among them 1-(S)-[3-hydroxy-2-(phosphonomethoxy)propyl]-5-azacytosine (HPMP-5-azaC,), its cyclic form (cHPMP-5-azaC) and ester prodrugs. From the structural point of view, HPMP-5-azaC is a 5-azacytosine analog of CDV, 1-(S)-[3-hydroxy-2-(phosphonomethoxy)propyl]cytosine [6,7].

## 2. Anti-poxvirus Activity *in vitro*

CDV was found to be effective against a broad range of DNA viruses, including adeno-, herpes-, irido-, hepadna-, papilloma-, polyoma- and poxviruses. All herpesviruses of human and veterinary importance are inhibited by CDV. Particularly important is the activity of CDV against: (i) adenoviruses for which there is currently no drug treatment; (ii) herpes simplex virus (HSV) and varicella-zoster virus (VZV) mutants resistant to acyclovir due to mutations in the viral thymidine kinase (TK); (iii) HCMV mutants with alterations in the viral UL97 gene, whose product is responsible for the activation of ganciclovir; (iv) Epstein-Barr virus (EBV), for which no antiviral treatment is available; (v) polyoma- and papillomavirus, for which also no therapy is currently approved; and (vi) poxviruses, including variola virus, which is considered as a possible bioterrorist weapon.

The *in vitro* activity of CDV against vaccinia virus (VACV) was first mentioned in the context of a comparative study with HPMPA. CDV was found to inhibit VACV replication *in vitro* at an IC_50_ of 4 μg/mL, while under the same conditions HPMPA showed an IC_50_ of 0.7 μg/mL against VACV [1]. Several studies later on confirmed the activity of CDV against VACV and enlarged the spectrum of activity of CDV against several orthopoxviruses [*i.e.*, cowpox (CPXV), camelpox (CMLV), monkeypox (MPXV), ectromelia virus (ECTV), and variola (VARV)]. In fact, from all the poxviruses evaluated for their susceptibility to the inhibitory effects of CDV, variola virus proved to be one of the most sensitive orthopoxviruses [8–11]. Further studies have ascertained that CDV is also effective against parapoxviruses, a group of viruses that cause orf in sheep and goats, pseudocowpox in cattle and skin lesions in deer, seals, squirrels and camels [12].

**Figure 2:**
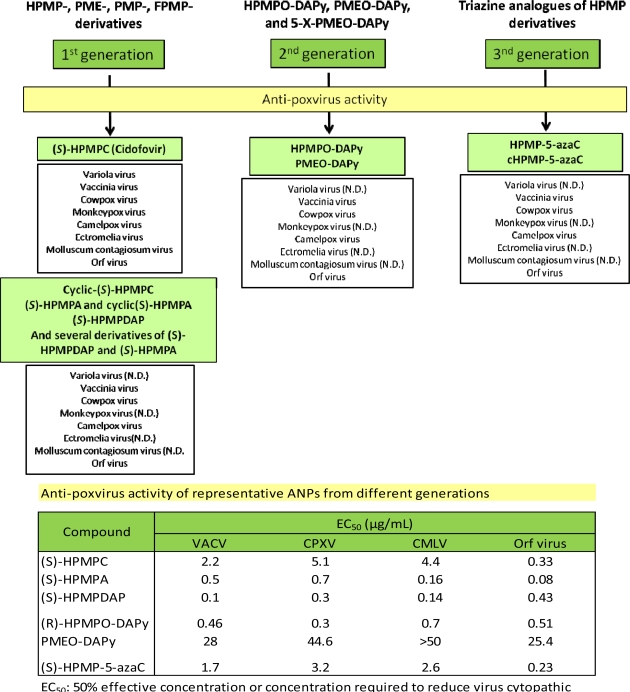
Spectrum of anti-poxvirus activity of CDV and other acyclic nucleoside phosphonates (ANP)s. The antiviral properties of ANPs others than HPMPC (CDV) against variola virus, monkeypox virus and molluscum contagiosum virus has not been determined yet. ND: not determined.

A summary of the activity spectrum against poxviruses described for the different generations of ANPs is given in Figure 2. Among the O-linked ANP analogues, HPMPO-DAPy was found to exhibit selective and potent activity against several poxviruses *in vitro*. Comparison of CDV and its 5-aza analog showed that anti-poxvirus activity and selectivity data of HPMP-5-azaC are similar, or in some cases higher, than CDV [6,7]. It should be noticed that for a number of the ANPs, the activity against certain poxvirus, such as ECTV, MPXV, VARV, and molluscum contagiosum virus (MCV) has not been evaluated yet.

Inhibition of poxvirus replication by CDV in combination with other agents has not been much investigated. Vigne *et al.* [13] found strong synergistic effects when CDV was combined with small interfering RNAs (siRNAs) targeting the D5R, B1R, or G7L genes that encode for, respectively, a DNA-independent nucleoside triphosphatase, a serine/threonine kinase, and a protein of the core of the intracellular mature virus.

At first, most of these experiments were performed in monolayer cell culture assays. More recently, VACV, CPXV, CMLV, and orf virus were shown to replicate efficiently in three-dimensional epithelial raft cultures, using human or lamb keratinocytes, giving histological pictures comparable to that described for the skin biopsy specimens of the corresponding diseases. In these conditions, CDV and several acyclic nucleoside analogs exhibited the expected selective antiviral activity [14–16].

## 3. Intracellular Metabolism

### 3.1. Cellular Uptake

The negative charge of the phosphonate moiety of the ANPs significantly impairs their cellular uptake. Their membrane transport is an active process and it is significantly slower and less efficient than that of nucleoside analogs, which can cross the cell membrane by the nucleoside transport carrier system or by passive diffusion. It has been suggested that the cellular uptake of ANPs occurs via an endocytosis-like process with slow kinetics and marked temperature dependence [17].

In a later study, Connelly and collaborators [18] studied the uptake of CDV into Vero cells and their data confirmed that the uptake of CDV was temperature sensitive: the rate of uptake was considerably lower at 27 °C than at 37 °C and almost totally inhibited at 4 °C. The uptake of [^3^H]CDV into Vero cells was compared to that of [^14^C]sucrose, an indicator for fluid-phase endocytosis. The uptake kinetics for both [^3^H]CDV and [^14^C]sucrose into Vero cells were very similar, as well as the effects of the microtubule antagonist colchicine (inhibitor of endocytosis) and of the tumor promoting agent phorbol myristate acetate (stimulator of endocytosis). It was, thus, concluded that CDV enters the cells via fluid-phase endocytosis and that once internalized it may accumulate in the lysosome. Protonation of the negative charge on the phosphonyl group in CDV may permit its diffusion across the lysosome membrane and then in the cell cytoplasm the compound is converted to the active diphosphorylated form.

### 3.2. Activation and Intracellular Half-life

**Figure 3:**
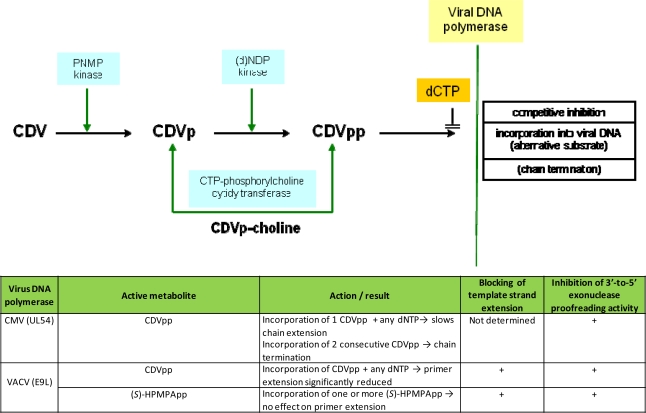
Mechanism of action of Cidofovir (CDV). Once inside the cells, CDV needs to be activated by cellular enzymes. Pyrimidine nucleoside monophosphate (PNMP) kinase catalyses the conversion of CDV (CDV) to CDV-monophosphoryl (CDVp), which is then further phosphorylated to the active form, CDV-diphosphoryl (CDVpp) by nucleoside 5′-diphosphate (NDP) kinase. CDVp-choline is considered to serve as an intracellular reservoir for the mono- and diphosphoryl derivatives of CDV. The diphosphoryl derivative of CDV (*i.e.*, CDVpp) interacts with the viral DNA polymerase as either competitive inhibitors [with respect to the natural substrates (*i.e.*, dCTP)] or alternative substrates (thus leading to incorporation into DNA). CDV has a hydroxyl function in the acyclic side chain that would allow further chain elongation. For human cytomegalovirus (HCMV), chain termination occurs when two consecutive CDVpp are incorporated in the growing DNA chain. The mechanism of action of CDV and (S)-HPMPA against VACV DNA polymerase compared to CMV polymerase was adapted from [27].

Once in the cytoplasm, CDV needs only two phosphorylation steps to be converted to its antiviral active diphosphoryl derivative, *i.e.*, CDV-pp (CDVpp) (Figure 3). The two phosphorylation steps are carried out by cellular enzymes [19]. In this way, CDV is independent of the first phosphorylation step, which in the acyclic nucleoside analogs (such as acyclovir and ganciclovir) is catalyzed by the HSV or VZV encoded TK or the HCMV encoded protein kinase UL97. Therefore, the compound is active against TK deficient HSV and VZV mutants and UL97 HCMV mutants. In a study performed by Bronson *et al.* [20], the metabolism of CDV was shown to remain unchanged between uninfected and infected cells, indicating that neither viral enzymes nor viral-induced enzymes are required for the activation of the compound.

Pyrimidine nucleoside monophosphate (PNMP) kinase catalyzes the first step of phosphorylation (CDV → CDVpp); whereas the second step (CDVp → CDVpp) is catalyzed by nucleoside diphopsphate (NDP) kinase, pyruvate kinase or creatinine kinase. CDVpp can be used by the choline phosphate cytidyl transferase to form the CDVp-choline adduct, according to the reaction: CDVpp + choline phosphate → CDVp-choline + pyrophosphate [21].

A general feature of CDV is the long intracellular half-life of the diphosphoryl metabolites, which allows infrequent dosing of the compounds. The long-lasting antiviral action of CDV may be attributed to the long half-life of the CDV metabolites (*i.e.*, CDVp, CDVpp and CDVp-choline) that are formed intracellularly following uptake of CDV by the cells. After removal of CDV from the cell culture medium, the intracellular levels of CDVp and CDVpp show a biphasic decline with half-lives of ∼24 h and ∼65 h [21,22]. This is most probably due to the accumulation of the CDVp-choline metabolite, which has a half-life of ∼87 h, and may be considered to be a reservoir or depot form for CDV [21].

## 4. Mechanism of Antiviral Activity

The antiviral effect of CDV is the result of a selective interaction of its diphosphoryl metabolite with the viral DNA polymerases; the specificity of CDV derives in part from a higher affinity of CDVpp for viral DNA polymerases than for host-cell polymerases. The binding affinity of CDVpp for HCMV DNA polymerase, as represented by the inhibition constant (Ki), is of 6.6 μM, which is approximately 8- to 80-times greater than for human DNA polymerases [Ki = 51 μM (DNA polymerase α), Ki = 520 μM (DNA polymerase β) and Ki = 299 μM (DNA polymerase γ)] [23–25]. The inhibition constants for CDVpp for other herpes viral DNA polymerases have also been determined; the Ki values against HSV-1 and HSV-2 polymerases are, respectively, 0.86 μM and 1.4 μM, providing a selective binding affinity of up to 600-fold for the viral enzymes [24].

How does CDVpp interfere with viral DNA synthesis? CDVpp can serve as a competitive inhibitor with respect to the natural substrate, *i.e.*, dCTP, or it can act as an alternative substrate and then be incorporated after removal of the pyrophosphate group [25]. Since CDV contains a hydroxyl function in the acyclic side chain, its incorporation does not inevitably result in chain termination.

The principles of the mode of antiviral action of CDV have been first studied for CMV. Xiong and colleagues [23,26] have indicated that HCMV DNA polymerase incorporates CDV into the DNA with correct fidelity and the fidelity of DNA elongation is maintained following the incorporated CDV. It is incorporated internally into DNA by HCMV DNA polymerase. Incorporation of a single molecule of CDV slows down HCMV DNA synthesis by 31%; incorporation of two molecules of CDV separated by one or two natural nucleotides drastically slows down DNA synthesis, whereas incorporation of two consecutive molecules of CDV completely prevents DNA elongation by HCMV DNA polymerase. HCMV DNA polymerase associated 3′ → 5′ exonuclease activity cannot excise CDV from the 3′ end due to the presence of the phosphonate group in the incorporated CDV molecule; and the rate of DNA synthesis slows down by 90% when using a DNA template that contains one internally incorporated CDV molecule. Thus, CDV can interfere with HCMV DNA synthesis in a number of ways, the most efficient being DNA chain termination following two consecutive incorporations of CDV at the 3′-end. The inhibition of HCMV DNA polymerase by CDVpp and the inability of HCMV DNA polymerase to excise incorporated CDV from DNA may account for the potent and long-lasting anti-HCMV activity of CDV [26].

The mechanism of inhibition of the E9L DNA polymerase of VACV is somewhat different from that of HCMV. Recently, Magee and collaborators have studied the effect of CDVpp and HPMPApp on VACV DNA polymerase [28,29]. CDVpp is a poor substrate for DNA synthesis relative to dCTP. CDVpp can be faithfully incorporated into primer strands by VACV DNA polymerase without a complete inhibition of further chain elongation. However, incorporation of consecutive CDVpp residues into the primer strand does impede elongation rates. The authors also showed that both CDVpp can be excised from the primer 3′ terminus by the 3′-to-5′ proofreading exonuclease activity of VACV polymerase, but DNAs bearing CDVpp as the penultimate 3′ residue are refractory to removal by VACV DNA polymerase’s 3′-to-5′ proofreading activity. More recently, an additional mechanism of action of CDV was discovered. Magee *et al.* showed that templates containing a CDV residue cannot be extended beyond the CDV base by the VACV DNA polymerase [29]; CDV creates a lesion that further blocks elongation by the VACV DNA polymerase and, thus, effectively blocks further rounds of replication.

When the mechanism of action of HPMPA diphosphate on the vaccinia E9L DNA polymerase was studied, some differences with CDVpp were seen [29]. Surprisingly, unlike CDVpp, (S)-HPMPApp is an excellent substrate for the E9L polymerase (K_m_ and V_max_ similar to that of dATP). (S)-HPMPApp is readily incorporated into the growing DNA strand and, unlike CDVpp, it does not slow chain extension but blocks 3′-to-5′ exonuclease activity when in the penultimate position (Figure 3). At the primer terminus, (S)-HPMPApp can still be excised. Similarly to CDVpp, when (S)-HPMPApp is incorporated into the template strand, it strongly inhibits trans-lesion DNA synthesis. If nucleotide analogues are incorporated into the template strand, they can severely inhibit polymerase activity, similarly to some forms of DNA damage. This mode of action is not relevant for most DNA polymerase inhibitors because the majority of them are obligate chain terminators. In the case of (S)-HPMPA, (S)-HPMPApp is a good substrate but not an effective chain terminator and it may act more by inhibiting secondary rounds of DNA synthesis. The relatively greater efficacy of (S)-HPMPA in comparison to CDV can be explained by a combination of factors related to higher intracellular levels of (S)-HPMPApp plus a superior likelihood that (S)-HPMPA would be incorporated into an irreparable DNA lesion. Since (S)-HPMPApp is readily incorporated into DNA and does not slow chain extension, many (S)-HPMPApp residues may be incorporated into the template strand and templates containing (S)-HPMPA cannot be extended, blocking further rounds of replication and leading to template strand inhibition [29].

Since CDV and (S)-HPMPA inhibited VACV DNA polymerase more severely when incorporated into the template strand, mostly affecting secondary rounds of DNA synthesis; they are expected to compromise different processes, including virus assembly. Jesus and collaborators have analyzed the effects of CDV on the replicative cycles of distinct VACV strains [30]. They showed that despite an approximately 90% inhibition of production of virus progeny, virus DNA accumulation was reduced only 30%, and late gene expression and genome resolution were unaltered. Electron microscopic analysis of virus-infected cells treated with CDV revealed a reduction in the number of mature forms of virus particles, along with an increase in the number of spherical immature particles. They detected inhibition of genome encapsidation and proteolytic processing of the precursors p4a and p4b, ultimately leading to the impairment of virus morphogenesis. However, these effects of CDV on virus morphogenesis resulted from a primary effect on virus DNA synthesis, which led to later defects in genome encapsidation and virus assembly. Analysis of virus DNA by atomic force microscopy revealed that viral cytoplasmic DNA synthesized in the presence of CDV had an altered structure, forming aggregates with increased strand overlapping not observed in the absence of the drug. These aberrant DNA aggregations were not encapsidated into virus particles. The authors hypothesized that the incorporation of CDV into DNA molecules by the viral DNA polymerase would be the first step towards the downstream effect of the drug on virus morphogenensis, possibly related to alterations of DNA structure and subsequent impairment of DNA encapsidation. Alterations in DNA structure induced by the incorporation of nucleoside analogues could compromise the interaction of DNA with DNA-binding proteins and the subsequent encapsidation of the viral genome.

Watanabe and Tamaki [31] have shown that CDVpp (at concentrations of 20–50 μM) was able to inhibit MCV DNA polymerase activity, providing support for CDV as a treatment for severe cases of molluscum contagiosum.

## 5. Mechanism of Resistance

So far, there is no conclusive evidence of selection of cytomegalovirus (or other viruses) resistant to CDV in patients receiving treatment with CDV. Clinical failure of CDV has not been related to the emergence of drug-resistant strains so far. Characterization of *in vitro* selected CDV resistant herpesviruses has clearly shown that CDV resistant is linked to mutations in the viral DNA polymerase.

Similarly to herpesviruses, exposure of poxviruses to increasing concentrations of CDV *in vitro* selects for drug-resistant viruses with mutations in the E9L gene (DNA polymerase) [32]. These mutations are located at the 3′-to-5′ exonuclease domain and the 5′-to-3′ polymerase domain of the VACV DNA polymerase. Independent studies found that VACV strains that have been passaged under increasing concentrations of CDV bear either an alanine-to-threonine or an alanine-to-valine change at position 314 in the viral DNA polymerase [33–35]. By marker transfer experiments it could be demonstrated that the A314T substitution could confer a 5-fold increase in CDV resistance in VACV compared to the wild-type virus [33]. Considering this residue’s location in the putative exonuclease domain of the viral polymerase, this substitution may alter the ability of the enzyme to remove CDV residues from the viral polymerase. The alanine-to-valine substitution at position 684 in the putative polymerase domain was also shown by marker rescue experiments to confer resistance to CDV independently of the A314T substitution, although the degree of resistance was significantly lower than the virus encoding both mutations [33]. Interestingly, the A314T recombinant virus and the A684V recombinant viruses showed differences in sensitivity to the pyrophosphate analogue PAA: the A314T mutation conferred hypersensitivity to PAA; the A684V substitution showed increased resistance to PAA, while the presence of both mutations resulted in no change in susceptibility to PAA [33].

**Figure 4:**
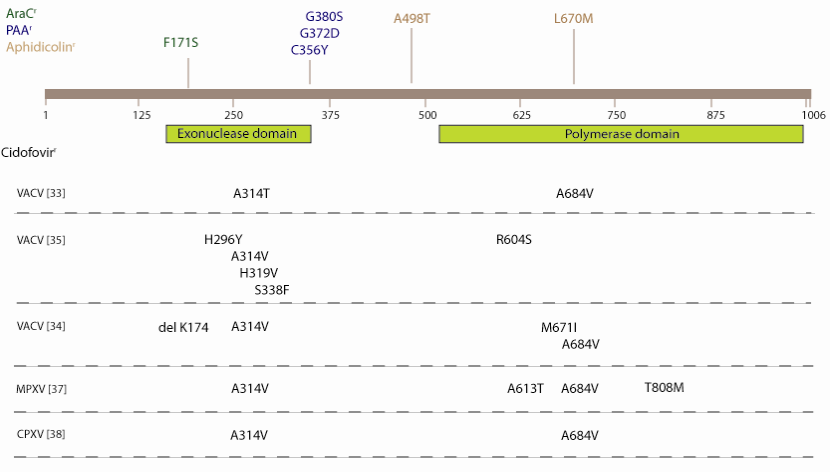
Location of mutations in CDV-resistant E9L (DNA polymerase) mutants.

Several other substitutions in the exonuclease domain and the polymerase domain in the VACV DNA polymerase of CDV-resistant viruses have been reported [34,35]. A summary of the different mutations identified in viruses isolated under selective pressure with CDV is presented in Figure 4. Becker *et al.* reported [34] that the A314V substitution is able to confer a seven-fold resistance to CDV and these viruses were shown to grow poorly in cell culture. In contrast, the virus bearing the A314T amino acid change proved to replicate as well as the wild-type in cell-culture. Thus, even if the level of resistance conferred by the two substitutions appeared to be equivalent, the viruses encoding the A314T substitution appeared to be more fit in cell culture than viruses encoding the A314V change. Consistent findings were found when the pathogenicity of the A314T and A314V mutants were evaluated in mice since both mutant viruses displayed impaired pathogenicity compared to the wild-type virus. Also the A684V and the double mutant A314 + A684V viruses exhibited reduced virulence in mice, demonstrating that these DNA polymerase mutations are linked to reduced fitness *in vivo* [33–35]. Interestingly, it was shown that infections caused by CDV^r^ VACV were still treatable with CDV treatment. It was observed that treatment for five days with CDV at 10 or 50 mg/kg once a day still protected mice against an intranasal challenge with the drug-resistant virus bearing both mutations [33].

In a recent study, it was demonstrated that the 3′-to-5′ proofreading exonuclease activity of VACV DNA polymerase is essential, and plays a key role in promoting genetic recombination [36]. In addition, a VACV DNA polymerase bearing the A314T substitution can overcome the inhibitory effects of CDV in both *in vitro* recombination and exonuclease assays. Thus, the A314T substitution enhanced the enzyme’s capacity to excise CDV molecules from the 3′ ends of duplex DNA and to recombine these DNAs *in vitro*, when experiments were performed using purified mutant DNA polymerase. Importantly, CDV was able to block the formation of concatemeric recombinant molecules *in vitro* in a process that was catalyzed by the proofreading activity of VACV DNA polymerase. Recombination was also inhibited when CDV-containing recombination substrates were transfected into cells infected with wild-type virus but not if transfected into cells infected with the virus bearing the A314T mutation mapping within the 3′-5′ exonuclease domain of the viral DNA polymerase.

Kornbluth and colleagues described a CDV^r^ VACV, which was found to encode five amino acid changes: four in the exonuclease domain (H296Y, A314V, H319W, S338F) of the VACV E9L polymerase and one in the polymerase domain (R604S) [35]. Transfer of this mutant E9L gene into wild-type VACV by marker rescue conferred the drug-resistance phenotype. However, the role of these mutations has not been clearly established because viruses encoding individual mutations were not isolated. E9L polymerase mutations occurred sequentially during passage in CDV, and an H296Y/S338F double mutant that conferred an intermediate CDV resistance phenotype was identified. *In vitro*, the marker-rescued CDV-resistant VACV containing all five mutations grew nearly as well as wild-type VACV. However, the virulence of this virus for mice was reduced, as 10- to 30-fold more CDV-resistant virus than wild-type virus was required for lethality following intranasal challenge. A single dose of CDV 50 or 100 mg/kg gave 60 to 80% survival *versus* 20% in untreated animals. Thus, independent investigations have shown that CDV^r^ VACV are less virulent in mice and despite a 9- to 14-fold *in vitro* resistance, the disease can be treated effectively with CDV.

In a recent study, a comparative whole genome sequence analysis of wild-type and CDV^r^ MPXV revealed 55 single-nucleotide polymorphisms (SNPs) and one tandem-repeat contraction [37]. Over one-third of all identified SNPs were located within genes comprising the poxvirus replication complex, including the DNA polymerase, RNA polymerase, mRNA capping methyltransferase, DNA processivity factor, and poly-A polymerase. Four mutations were found in the DNA polymerase gene, including the A314V and A684V mutations. The significance of the two other mutations in the viral DNA polymerase (A613T and T808M) on CDV resistance is not known. Also, both the A314V and A684V mutations have been reported in a cowpox CDV^r^ strain [38]. These data suggest that the mechanism of CDV resistance may be highly conserved across orthopoxviruses.

It should be noted that in the process of selection of CDV^r^ mutants, the A684V mutation appeared to be selected after the A314T mutation and that the A314T/V mutation has also been reported in viruses selected for resistance to other ANPs [33]. In fact, the A684V substitution has not been reported to occur alone following selection with either CDV or other ANPs; it has systematically been found in combination with other changes in the DNA polymerase of orthopoxviruses. The A684V mutation in combination with the S851Y substitution has been found in a VACV strain selected under pressure with (S)-HPMPDAP [39]. A recombinant virus bearing only the S851Y mutation exhibited a low level of resistance to dCMP analogues but high-level resistance to dAMP analogues and to 6-[3-hydroxy-2-(phosphonomethoxy)propoxy]-2,4-diaminopyrimidine, which is considered to mimic the purine ring system. The S851Y virus showed a reduced fitness *in vitro* and *in vivo*.

## 6. *In Vivo* Efficacy in Animal Models for Poxvirus Infections

Several animal models using mice (most frequently), rabbits, or monkeys have been used to demonstrate the activity of CDV against orthopoxvirus infections (reviewed in [40,41]). The treatment of VACV infections have been well studied in models involving infection of scarified skin, or resulting from intravenous, intraperitoneal, intracerebral, or intranasal virus inoculation. CPXV has been used in intranasal or aerosol infection studies to evaluate the treatment with CDV of lethal respiratory infections. Monkeypox, ectromelia, and variola viruses have been employed to a lesser extent than the other viruses. Also, the efficacy of topical CDV against orf virus in lambs has been described [42]. A summary of the efficacy of CDV in different models of poxvirus infections in presented in Table 1.

An interesting study has compared the effectiveness of post-exposure smallpox vaccination and antiviral treatment with CDV or with the related analogue HPMPO-DAPy after intratracheal infection of cynomolgus monkeys with MPXV [43]. Beginning antiviral treatment 24 h after lethal intratracheal MPXV infection with either drug following various treatment regimens resulted in a significant reduction in the number of cutaneous MPXV lesions and in mortality. In contrast, vaccination of monkeys 24 h after MPXV infection did not result in reduction of mortality, indicating that antiviral treatment with CDV is more effective than smallpox vaccination upon lethal MPXV infection.

Another study has evaluated the effects of coadministration of CDV and smallpox vaccine in monkeys [44]. The data indicated that a single-dose vaccination regimen including the smallpox vaccine Dryvax and CDV reduced VACV loads after vaccination and Dryvax-mediated vaccination complications. However, coadministration of CDV + Dryvax also significantly decreased Dryvax-elicited antibody and T-cell responses and impaired Dryvax-induced immunity against MPXV.

## 7. Dosage and Administration

CDV has been approved and marketed worldwide (Vistide®) for the treatment of HCMV retinitis in AIDS patients. The compound has to be given intravenously at a dose of 5 mg/kg once weekly for two weeks followed by 5 mg/kg intravenously once every other week. Strict monitoring of renal function before initiation of CDV therapy and concomitant administration of oral probenecid and intravenous hydration are required to minimize drug-related nephrotoxicity. The compound has been used off-label topically as a 3% or 1% cream formulated in different bases. No oral formulations are currently available.

Since the use of CDV is limited by its poor oral bioavailability and renal toxicity, Hostetler’s group has synthesized alkoxyalkyl esters of CDV and its cyclic form, *i.e.*, c-CDV [45–47]. Esterification of cidofovir with an alkoxyalkyl group facilitated drug adsorption in the gastrointestinal tract. These alkoxyalkyl esters of CDV and its cyclic form were much more active *in vitro* than the parent compounds against several herpesviruses, and poxviruses. The increased activity of alkoxyalkyl esters of CDV compared to the parent compound CDV was also shown against adenovirus, polyomavirus, and papillomavirus [48–50]. In addition, these derivatives showed improved uptake and absorption, and had oral bioavailabilities in mice of 88–97%, compared to less than 5% for CDV. Studies with radiolabeled compound confirmed increased cell penetration (10–20 fold) and higher intracellular levels (100-fold) of diphosphorylated CDV (the active form of the compound) than those measured following treatment of the cells with CDV [51]. *In vivo*, oral administration of the hexadecyloxypropyl- CDV (HDP-CDV) proved as effective as parental CDV in the treatment of herpes- and poxvirus infection in several mouse models [52–54]. Importantly, diminished accumulation of the drug in the kidney was reported according to studies evaluating tissue distribution of radiolabel HDP-CDV and other alkoxyalkyl esters of CDV in mice [55,56]. If no accumulation of these prodrugs is also observed in the clinic, these compounds may avoid the dose-limiting toxicity of CDV.

HDP-CDV (CMX001) in an oral formulation is presently under development by Chimerix. A Phase I clinical study to evaluate the safety and pharmacokinetics of orally administered CMX001 in healthy volunteers is completed and Phase II trials are ongoing in CMV infections in stem cell transplant recipients and polyoma BK virus infection in kidney transplant patients. CMX001 is also under consideration to be included in the Strategic U.S. Stockpile for emergency use in case of a bioterrorist attack with VARV or for treatment of smallpox vaccination in case massive vaccination should be required.

## 8. Pharmacology

The pharmacokinetic properties of CDV in humans have been reported for the intravenous preparation; CDV exhibited dose-independent pharmacokinetic features [57]. The clinical pharmacokinetic properties of CDV following single intravenous infusion at the recommended dosage regimen (5 mg/kg, with concomitant oral administration of probenecid) have been reviewed based on data compiled from three Phase I/II studies in HIV infected patients without CMV infection or with asymptomatic retinitis: C_max_ (peak serum concentration) of 19.6 ± 7.18 mg/L; AUC_0 → ∞_ (area under the serum concentration-time curve from zero to infinity) of 40.8 ± 8.97 mg/L h; creatinine clearance (based on serum creatinine concentration) of 0.138 ± 0.036 L/h/kg; renal clearance of 0.096 ± 0.031 L/h/kg; steady state volume of distribution of 0.39 ± 0.13 L/kg; and a plasma elimination half-life (t1/2β) of 2.2 ± 0.5 h) [58].

It should be noted that conventional pharmacokinetic measurements do not accurately reflect the duration of action of CDV, since the antiviral effect is dependent on the intracellular concentrations of the active phosphorylated metabolites within cells [59]. As mentioned above, the metabolites of CDV have a long intracellular half-life (48 h for the CDVp-choline adduct), which may contribute to the prolonged antiviral action of CDV.

Brody and colleagues reported on the pharmacokinetic properties of CDV in patients with renal insufficiency [60]. A significant correlation was observed between creatinine clearance and CDV clearance in patients with varying degrees of renal insufficiency; indicating the necessity to adjust the CDV dose in patients with kidney disease to ensure comparable drug exposure based on serum levels. Although CDV is contraindicated in subjects with renal impairment function, in this study, the authors provide theoretical dosing guidelines for this population of patients with reduced doses of CDV that would produce the required systemic exposure to CDV.

A limited amount of data is available concerning the systemic distribution of CDV following intralesional or topical application. CDV serum dosages were reported in a study performed to evaluate the efficacy of intralesional injections of CDV in patients suffering from severe laryngeal papillomatosis [61]. The patients received different numbers of CDV injections, and a total of 121 CDV injections were undertaken in a total of 17 patients (drug concentration = 2.5 mg/mL). Five series of CDV serum dosages were performed in three different patients. The drug could be detected in two patients: in one case the concentration of CDV in serum was 0.36 μg/mL 10 min after injection; in the second case the serum dosages were done on three different occasions and the drug could be detected at 5, 10, and 15 min after the end of injection; the concentrations of CDV were 0.59, 0.60, and 0.42 μg/mL, respectively. For the third patient and the two other series of serum dosages, the passage of CDV in the bloodstream, from the intralesional injections, could not be demonstrated.

In a later study, a linear relationship between CDV plasma concentration and dose in children, but not in adults, was found following local CDV injections in respiratory papillomatosis [62]. The same relationships were found between dose and area under the concentration/time curve (AUC). From this study, it was concluded that the CDV plasma levels were below those leading to toxicity and the levels and the AUC were dose dependent in children but not in adults. Diffusion from the injected site was the greatest and unpredictable among adults. Due to the great individual variation in diffusion in adults, it is recommended to use CDV at a lower dose than the recommended intravenous dose to prevent any risk of systemic toxicity [63].

No studies have been performed on CDV distribution following topical administration of the drug. The bioavailability and metabolism of CDV was studied in New Zealand white rabbits following topical administration to normal and abraded skin [64]. Concentrations in kidney following topical administration of CDV to normal skin were <4% of those following intravenous dosing. Topical application of CDV to intact skin led to negligible systemic exposure to the drug. However, the topical bioavailability and hence the flux of CDV through intact skin, was enhanced in abraded skin. Thus, abrasion of the skin removed the principal barrier to absorption and led to significant systemic exposure to CDV; therefore, it is recommended to take systemic exposure following application of the drug to abraded skin into account.

## 9. Safety

Most toxicity issues associated with CDV are related to intravenous use of the drug. CDV has been associated with nephrotoxic side-effects in various species, *i.e.*, mice, rats, guinea pigs, rabbits and monkeys. Guinea pigs appeared to be particularly susceptible to the nephrotoxic effects of CDV [65,66]. Nephrotoxic side effects of CDV were also observed during the initial clinical studies with CDV in HCMV-infected patients. Nephrotoxicity associated with CDV manifested by proteinuria, glycosuria, and decreases in serum phosphate, uric acid, and bicarbonate, increases in serum creatinine, and degeneration and necrosis of the proximal renal tubule cells [67–69].

The nephrotoxicity associated with CDV is explained by the fact that the active uptake of the compound at the basolateral membrane of the kidney proximal tubular cells is faster than the efflux of CDV at the luminal-side (*i.e.*, into the urine), thus resulting in the accumulation of CDV or its metabolites in the renal tubular cells [70]. The human organic anion transporter 1 (hOAT1) was shown to interact with CDV as well as other acyclic nucleoside phosphonates. hOAT1 proved to play a critical role in the organ-specific toxicity of CDV [70–72] Recently, a study was conducted to investigate whether the other renal organic anion transporter hOAT3 and organic cation transporter hOCT2 transport these antivirals. The hOCT2 did not increase uptake of the antivirals; furthermore CDV (as well as adefovir and tenofovir) are substrates of hOAT3 as well as hOAT1, but quantitatively hOAT1 appeared to be the major renal transporter for acyclic nucleoside phosphonates [73].

In addition to intravenous hydration, the accumulation of CDV in the renal tubular cells can be prevented by an infrequent treatment schedule and the co-administration of probenecid, an inhibitor of organic anion transport that interferes with the transporter-mediated tubular uptake of CDV [74]. Oral probenecid coadministration has been shown to effectively protect monkeys receiving chronic intravenous CDV. In addition, to avoid nephrotoxicity, CDV should not be dosed higher than 5 mg/kg (intravenously) once weekly for two weeks, followed by 5 mg/kg every other week. The infrequent dosing of CDV, which still affords a significant antiviral activity, is based on the long lasting activity of the diphosphorylated metabolite of CDV.

No systemic side effects have been reported upon topical or intralesional CDV administration, although local reactions (*i.e.*, inflammatory response) may occur at the application site [75,76]. In a phase II double-blind placebo-controlled study, CDV 1% topical gel was shown to be effective in the treatment of anogenital warts, the side effects in the CDV- and placebo-treated groups being comparable [77].

Inflammation and/or erosion may occur at the site of application of CDV cream in patients [78]. Such erosions invariably heal and may actually reflect an effective response of the viral lesions to CDV treatment, although care should be taken not to apply CDV on abraded skin. Also, topical application should not exceed a concentration of 1%. If applied at a too high concentration over a too large surface of abraded skin, there is a risk of systemic toxicity, as illustrated by a case of acute renal failure in a bone marrow transplant recipient who was treated with topical CDV 4% for 12 consecutive days [79].

## 10. Clinical Efficacy of CDV in the Treatment of Poxvirus Infections

CDV is licensed for the treatment of CMV retinitis in AIDS patients; however, it has been used off-label for the treatment of infections caused by other herpesviruses and several DNA viruses, *i.e.*, polyoma-, papilloma-, adeno-, and poxviruses. CDV has great potential for the therapy and prophylaxis of poxvirus infections, including orthopox (smallpox, monkeypox, cowpox and vaccinia), parapox (orf) or molluscipox (molluscum contagiosum). Also, CDV appears particularly indicated for the treatment of complications arising from vaccination with the life vaccinia vaccine in immunocompromised patients (*i.e.*, progressive disseminated vaccinia, vaccinia gangrenosa, *etc*.) and other patients (*i.e.*, accidental vaccinia, eczema vaccinatum, *etc*.) in case smallpox vaccination should become necessary again [80,81]. Vaccination of immunosuppressed patients would be absolutely contraindicated, but inadvertent use of the life vaccine in this group of patients may lead to a serious, life-threatening disseminated and progressive vaccinia. Based on the data obtained in experimental animal models, CDV may be expected to be effective in the therapy as well as the pre- and post-exposure prophylaxis of smallpox, monkeypox and vaccinia virus infections in humans. Recently, a dramatic case of eczema vaccinatum in a child following infection from his father who has been vaccinated for smallpox was reported. The child was successfully treated with Vaccinia Immuno Globulin (VIG) and two antiviral agents, *i.e.*, CDV and ST-246, an anti-poxvirus drug that inhibits the morphogenesis of orthopoxviruses [82]. CDV was administered on the basis of standard induction dosing for patients with AIDS who have CMV retinitis; and only one dose was given because of clinical improvement over the next week. Due to the close timing of administration of each agent, it is difficult to precisely determine the contribution of each agent to the patient’s recovery. The levels of VACV DNA in the blood started to decrease following VIGIV and CDV treatment, and then continued to decrease following ST-246 administration [83].

Monkeypox has been considered as an emerging zoonosis after the virus was introduced into the U.S., and infected prairie dogs had contaminated clusters of patients in the Midwest [84–86]. While there is no clear clinical data on the usefulness of CDV in such patients, CDV can be used in case of emergence of a monkeypox outbreak since CDV was found to decrease morbidity and mortality associated with monkeypox infections in two animal models (Table 1). Primates treated with a single dose of CDV on the day of infection were completely protected from clinical and laboratory signs of disease.

Recently, several outbreaks of cowpox in domestic animals and humans have been reported in Europe [87–92]. Cowpox infections in humans can lead to massive local destruction. This can lead to mutilation and, depending on anatomic site, significant functional disability and also aesthetic disfigurement. Considering the efficacy of CDV in different animal models of CPXV infection, CDV is a promising therapeutic option for the treatment of emerging CPXV zoonosis in humans.

CDV has been used in the clinic to treat patients with recalcitrant molluscum contagiosum. Meadows *et al.* reported three HIV seropositive patients with extensive molluscum contagiosum, two of whom received intravenous CDV and one of whom received topical CDV [93]. The two patients receiving intravenous CDV received the drug for the treatment of CMV retinitis and over one or two months therapy a complete resolution of the molluscum contagiosum lesions were noted. The lesions completely regressed in the patient receiving CDV 3% (once a day, Monday through Friday for two weeks) after one month therapy. Moderate inflammation appeared during the second week of therapy, but one month following therapy, neither molluscum contagiosum lesions nor residual inflammation were noted. A complete and durable resolution of molluscum contagiosum lesions was observed in children presenting extensive lesions following topical application of CDV 1% or 3% cream, e.g., in a boy with Wiskott-Aldrich syndrome [94], in two otherwise healthy children [95] and in two HIV-seropositive children [96]. Topical CDV (3% cream) in combination with cryotherapy has proved to be efficacious in the treatment of a giant facial molluscum contagiosum in an HIV patient [97]. After three months of treatment, more than 70% improvement in the clinical picture was observed, with a marked improvement after 12 months of combination therapy. Two years after discontinuation of all topical treatment and despite no improvement in immune status, the patient remained free of mollusci.

The first report on the efficacy of CDV in the treatment of a giant orf (ecthyma contagiosum) lesion in an immunosuppressed patient was described in 2001 [98]. Topical treatment with 1% CDV cream (five cycles of 5 days with and 5 days without treatment) resulted in complete resolution of the lesion, with only granulation left. After some signs of recurrence, the lesion was treated with another two courses of CDV cream, affording a complete cure. However, in a recent case-report of a 73-old woman with non-Hodgkins lymphoma who developed progressive orf virus lesions, CDV administered topically and intralesional did not result in resolution of the lesions [99]. The patient’s orf virus infection regressed with topical imiquimod despite progression of her malignancy.

## 11. Conclusions

CDV remains a reference compound against poxviruses and holds potential for the therapy and short-term prophylaxis of poxvirus infections. Unlike ST-246, an inhibitor of orthopoxvirus morphogenesis, CDV can be used not only for the treatment of orthopoxviruses (smallpox, monkeypox, cowpox, vaccinia), but also of parapox (orf) and mollusci (molluscum contagiosum) viruses. Treatment of poxviruses infections should be considered not only in the case of an eventual bioterrorist attack with smallpox, but also poxviruses zoonosis (*i.e.*, cowpox, monkeypox, and orf), considering the increasing numbers of reports of outbreaks. Also, the veterinary use of CDV should not be neglected. Furthermore, the management of molluscum contagiosum infections in children and in immunocompromised patients can be difficult and may require the use of effective antiviral agents. Also, the potential of novel derivatives of CDV (such as HPMP-5-azaC) as antipoxvirus agents, as well as CDV prodrugs (such as CMX001) for systemic treatment of poxviruses infections should be considered.

## Figures and Tables

**Table 1:** Efficacy of CDV in different models of poxvirus infections.

**Animal model**	**Route of CDV administration**	**Evidence for Efficacy**	**Reference**
Intranasal or aerosolized CPXV infection in mice	Subcutaneous	One inoculation of 100 mg/kg CDV on day 0, 2, or 4 resulted in 90–100% survival. Treatment on day 0 reduced peak pulmonary virus titers 10- to 100-fold, reduced the severity of viral pneumonitis, and prevented pulmonary hemorrhage. The same dose on day -6 to 2 protected 80%–100% of infected mice, whereas one inoculation on day -16 to -8 or day 3 to 6 was partially protective.	[100]
		A single dose of CDV 100 mg/kg administered on the day of infection was 80 to 100% protective when given on day 0, 1, 2, 3, or 4 after infection. Lung virus titers (determined on day 4 of the infection) were significantly reduced in groups treated on day 0, 1, or 2.	[101]
	Intranasal	Single treatment of 20 and 40 mg/kg CDV given up to three days after virus inoculation resulted in 80–90% protection. A single 40 mg/kg treatment of infected mice given 1 or 2 days after infection significantly decreased virus titer in lungs and nose/sinus compared to the placebo group.	[102]
		Single treatment of 5–40 mg/kg 24 h after virus exposure afforded 80–100% protection from lethal infection and significant reduction in viral titers in the lung tissue.	[103]
	Aerosol	Single treatment of 1-5mg/kg CDV at 1 day before or 2 h after infection showed efficacy as measured by changes in body and lung weight, lung viral titers, pulmonary pathology and survival.	[104]
		Treatment with CDV was successful in protecting against lethal intranasal cowpox infection. A dose of drug in the range of 0.5–5 mg/kg was protective when given before (day -1), the day of infection (day 0) or after infection (day +1 or +2); an 80% survival rate was observed when mice were treated 2 days before challenge.	[105]
	Intraperitoneal	Treatment for five consecutive days starting 24 h after infection with CDV at 30 mg/kg per dose was 100% effective in preventing mortality.	[106]
		Treatment with CDV at 160, 80 or 40mg/kg as a single dose 24 h after virus exposure afforded 100% protection from lethal infection and significant reduction in viral titers in the lung tissue.	[103]
		Treatment with CDV at 6.7 mg/kg once daily for five days beginning 24 or 48 h after viral inoculation, afforded 100% protection from lethal infection. Even when treatment was started 72 h post-infection, CDV treatment resulted in 66% protection.	[107]
		CDV at a dose of 5 or 10 mg/kg administered daily beginning on day -5, -3, or -1 through day 0 (the viral inoculation day) afforded 93% protection from lethal infection. A single dose of 30 mg/kg CDV -1 before viral inoculation or 1 day post-infection resulted in 100% protection.	[107]
		CDV at 100 mg/kg once a day on days 1 and 2 after infection resulted in 100 protection from lethal infection and significant reduction in viral titers in the lungs.	[108]
		CDV (100 mg/kg/day for two days starting 24 h after virus exposure) led to survival and suppression of tissue virus titers in animals suffering from either a lethal upper respiratory tract infection or both upper and lower respiratory tract infection.	[109]
Intranasal or aerosolized VACV infection in mice	Intraperitoneal	Treatment with CDV at 160, 80 or 40mg/kg as a single dose 24 h after virus exposure afforded 70% protection from lethal infection.	[103]
		Treatment with CDV at 5 mg/kg once daily for five days beginning 24, 48, or 72 h after viral inoculation, afforded 73–100% protection from lethal infection.	[107]
		CDV treatment (100 mg/kg/day i.p. for two days) significantly reduced mortality and viral titers in lungs.	[108]
	Intranasal	Single treatment of 5–40 mg/kg 24 h after virus exposure afforded 70–80% protection from lethal infection and significant reduction in viral titers in lung tissue.	[103]
Intraperitoneal CPXV infection in mice	Intraperitoneal	CDV at 100 mg/kg once a day on days 1 and 2 after infection resulted in significant protection from lethal infection and significant reduction in viral titers in the lungs.	[108]
		CDV at 25 or 100 mg/kg once one day before infection resulted in significant reduction of virus replication in several organs.	[110]
Intraperitoneal VACV infection in mice	Intraperitoneal	CDV treatment (100 mg/kg/day i.p. for two days) afforded 60% protection from lethal disease and significant reduction in viral titers.	[108]
Intravenous, intranasal, or intraperitoneal VACV infection in SCID mice	Subcutaneous	Following administration of CDV, at doses ranging from 1mg/kg/day for five days to 20 mg/kg/twice a week, death could be significantly delayed.	[111]
Intranasal CPXV infection in SCID mice	Subcutaneous	Treatment every three days with CDV (100 mg/kg) through day 30 of the infection resulted in significant delay in the time of death but final mortality	[101]
		Treatment with CDV at 100 mg/kg/dose starting on day 0 and repeating the dose every three days resulted in delay of time of death but not in protection from lethal infection.	[100]
Cutaneous CPXV infection in hairless mice	Intraperitoneal	Hairless mice treated with 50 mg/kg beginning +24 h after viral inoculation, 3× weekly for one week, had significantly reduced lesion-day AUCs (area under the curve) and mean peak lesion scores.	[112]
Cutaneous VACV infection in hairless mice or athymic nude mice	Intraperitoneal / topical	Hairless mice treated with 50 mg/kg of CDV (starting 24 h post-inoculation of the virus once a day for seven days) or topically with 5% CDV 3× a day for seven days) had a significantly lower lesion-day AUCs (area under the curve) and mean peak lesion scores.	[112]
		Topical treatment with 1% CDV, initiated at the day of infection or at day 1 p.i. during 5 days, completely protected against virus-induced cutaneous lesions and against associated mortality. Systemic treatment with CDV (100 mg/kg 3× or 5× per week initiated at 14 days post-infection caused healing and regression of the lesions.	[113]
		CDV at 100 mg/kg once a day on days 1 and 2 after infection resulted in protection from lethal infection and significant reduction in viral titers in the lungs.	[108]
		Topical treatment with 1%-CDV cream (twice daily for seven days) of immunocompromised mice (hair-less mice treated with cyclophosphamide) was much more effective in reducing the severity of primary lesions and the number of satellite lesions than was systemic CDV treatment (100 mg/kg/day, given every three days). Both forms of treatment delayed death. Topical drug treatment markedly reduced virus titers in the skin and snout, whereas systemic treatment did not.	[114]
Smallpox vaccine in monkeys	Intravenous	Coadministration of CDV (20 mg/kg) and smallpox vaccine reduced vaccination side effects but interfered with vaccine-elicited immune responses and immunity.	[44]
Footpad ECTV inoculation in mice	Intraperitoneal	Mice given 5 mg/kg/dose starting 24 h after infection had mild disease (reduced inflammation and footpad swelling) but showed a 100% recovery. Animals receiving higher doses of CDV (20 or 100 mg/kg/day) had mild footpad swelling and 100% recovery.	[115]
		Daily treatment with 100 mg/kg/day CDV for five days starting one day after infection with a mouse interleukin-4 (producing virus causing host immune dysfunction and severe disease) delayed but could not prevent death from systemic infection.	[115]
Intranasal ECTV inoculation in mice	Intraperitoneal	CDV injection at 5 mg/kg on day zero and at 1.25 mg/kg on day three protected 100% of animals from lethality.	[116]
Aerosolized MPXV infection in monkeys	Intravenous	A single treatment of 5 mg/kg on the day of infection resulted in significantly reduced mortality and completely protected the animals from clinically and laboratory signs of disease.	[117]
Intratracheal MPXV infection in monkeys	Intraperitoneal	A dose of CDV of 5 mg/kg every other day for five days or six doses starting one day after infection resulted in significantly reduced mortality and reduced numbers of cutaneous monkeypox lesions.	[43]
Intravenous MPXV infection in monkeys	Intravenous	5 mg/kg of CDV given before or up to two days after infection led to complete protection with no signs of illness and control of viral replication in blood.	[118–120]
Intravenous VARV infection in monkeys	Intravenous	5 mg/kg of CDV given before or up to two days after infection led to complete protection with no signs of illness and control of viral replication in the blood.	[118–120]
Hind thighs orf virus scarification in lambs	Topical	1% CDV given for four consecutive days resulted in milder lesions that resolved more quickly than untreated lesions. The scabs of the treated animals contained significantly lower amounts of viable virus meaning there should be less contamination of the environment with virus than would normally occur.	[42]
		Animals were treated with a paint of 0.5% or 1% CDV + sucralfate 15% (wound healing properties) + NaH2PO4 16% w/w and with sucralfate gel suspension alone as control. The treatment with formulations containing CDV and phosphate salt for four consecutive days resulted in a rapid resolution of the lesions, with scabs containing significantly lower amounts of viable virus when compared with untreated lesions and lesions treated with sucralfate suspension alone.	[121]
